# Use of HIV Phylogenetic Analysis in Public Health in Victoria, Australia: Regulatory Considerations

**DOI:** 10.1007/s11673-025-10496-7

**Published:** 2026-02-17

**Authors:** Casey M. Haining, Aaron Cogle, Richard Keane, George Taiaroa, Jane Brophy, Mihaela Ivan, Doris Chibo, Jane S. Hocking, Deborah A. Williamson, Sharon R. Lewin, Louise Keogh

**Affiliations:** 1 Centre for Health Equity, School of Population and Global Health, 207 Bouverie Street, Carlton VIC, Melbourne, 3053 Australia; 2 National Association of People with HIV Australia (NAPWHA), Suite G5 1 Erskineville Road, Newtown, NSW 2042 Australia; 3 Living Positive Victoria, Coventry House, Ground Floor, 95 Coventry Street, Southbank, VIC 3006 Australia; 4 Department of Infectious Diseases, The University of Melbourne at the Peter Doherty Institute for Infection and Immunity, 792 Elizabeth Street, Melbourne, VIC 3000 Australia; 5 Department of Health, Victoria, 50 Lonsdale Street, Melbourne, VIC 3000 Australia; 6 Victorian Infectious Diseases Reference Laboratory, The Royal Melbourne Hospital at the Peter Doherty Institute for Infection and Immunity, 792 Elizabeth Street, Melbourne, VIC 3004 Australia; 7 Centre for Epidemiology and Biostatistics, Melbourne School of Population and Global Health, 207 Bouverie Street, Carlton, VIC 3053 Australia; 8 Department of Infectious Diseases, Alfred Hospital and Monash University, 85 Commercial Road, 3004 VIC Prahran, Australia; 9 Victorian Infectious Diseases Service, The Royal Melbourne Hospital at the Peter Doherty Institute for Infection and Immunity, 792 Elizabeth Street, VIC 3000 Melbourne, Australia; 10 MRC–University of Glasgow Centre for Virus Research, Glasgow, G61 1QH United Kingdom

**Keywords:** HIV, Phylogenetic analysis, Molecular epidemiology, Public health, Criminalization, Regulation

## Abstract

Human immunodeficiency virus (HIV) phylogenetic analysis has been introduced in a select number of jurisdictions globally for the purposes of HIV public health surveillance. Using this epidemiological tool in this way can enhance public health efforts aimed at managing HIV transmission. This application, however, presents many legal and ethical concerns that may counter such efforts, particularly in regulatory environments where HIV transmission can be subject to criminal prosecution. This article uses Victoria, Australia, as a case study and analyses the current regulatory frameworks relevant to HIV transmission and explores how these may influence the future implementation of phylogenetic analysis in this setting. The article concludes by offering a series of regulatory recommendations including considerations relevant to law and policy reform; procedures and practice; education and guidance; and community engagement and research.

As of 2022, an estimated 39 million people were living with human immunodeficiency virus (HIV) (WHO 2023). While there is currently no cure, significant advances in HIV treatment have resulted in treatment combinations that are simpler, more tolerable, safer, and more efficacious than earlier treatments (Cihlar and Fordyce 2016). Such advances mean that the life expectancy of a person with HIV receiving antiretroviral therapy (ART) is now similar to that of a person without HIV, and HIV is no longer regarded as the death sentence it once was (Barré-Sinoussi et al. 2018; Boyd et al. 2016; Trickey et al. 2023).

People living with HIV who access ART and maintain a low viral load in their blood (<200 copies/ml) are unable to transmit the virus to others (Broyles et al. 2023).^1^ In addition, HIV-negative individuals can now take medication—either orally (tablet) or in an injectable form—to effectively prevent them from acquiring HIV (referred to as pre-exposure prophylaxis (PrEP)) (Grant et al. 2010; Landovitz et al. 2021; Molina et al. 2015). Despite such progress, HIV remains a public health concern due to ongoing transmission within priority groups, the emergence of drug resistance, and the challenges of diagnosis associated with PrEP (WHO 2023; Williams et al. 2023). To address this, the United Nations Programme on HIV/AIDS (UNAIDS) has set ambitious targets to accelerate the delivery of high-impact HIV prevention and treatment services, seeking to end the Acquired Immunodeficiency Syndrome (AIDS) epidemic as a public health threat (UNAIDS 2021). The 95-95-95 target calls for 95 per cent of all people living with HIV to know their HIV status, 95 per cent of those diagnosed with HIV infection to receive sustained ART, and 95 per cent of those receiving ART to achieve viral suppression by 2030 (UNAIDS 2021).

In Australia, an estimated 28,870 people were living with HIV at the end of 2022, 7 per cent of whom were unaware of their HIV status (Health Equity Matters 2024). At the end of 2021, “91% of people living with HIV were diagnosed, 92% of those diagnosed were on treatment and 98% of those on treatment were virally suppressed” (UNAIDS 2023 ¶4). Such outcomes are attributable to several public health interventions introduced in Australia to manage HIV transmission, ranging from needle and syringe programmes, testing incentivization efforts, rapid initiation of ART, expansion of PrEP prescribers, targeted sexually transmitted infection (STI) prevention strategies, HIV-related funding programmes and partnerships, and contact tracing (Obeng et al. 2024). Over time, HIV notifications have declined among certain groups, such as Australian-born men who have sex with men (MSM) (Medland et al. 2018). However, transmission remains a problem among other groups, including Asian-born MSM, heterosexual men and women, migrants, and Aboriginal and/or Torres Strait Islander peoples (Di Giallonardo et al. 2023; Medland et al. 2018).

Identifying individuals and connecting them with appropriate care and support is essential for reducing transmission in particular populations. While timely contact tracing may be somewhat of an effective method, existing approaches can be strengthened by integrating traditional epidemiological data with phylogenetic analysis (also known as molecular epidemiology) (Taiaroa et al. 2024). Phylogenetic analysis is a form of epidemiological typing that enables comparison of HIV genetic sequences from different sources and analysis of evolutionary relationships between them (Dawson et al. 2020). This can provide more granular detail of transmission patterns and assist in identifying clusters at an earlier stage than traditional methods (Taiaroa et al. 2024; Faber et al. 2025). Such techniques can serve as complementary surveillance tools to inform existing public health interventions (Taiaroa et al. 2024).

Despite this potential public health utility, the use of phylogenetic analysis raises several ethical and legal concerns, particularly in regulatory environments where alleged, perceived, or actual HIV transmission can be sanctioned (Nkengasong and Ratevosian 2023). Such settings risk reinforcing systemic stigma, discrimination, and marginalization —issues experienced by people living with HIV more acutely than those associated with many other viral infections (including other STIs). Significant information gaps within conventional detection strategies, including a reluctance to undergo screening or disclose sexual partners, have been attributed to HIV-related stigma (Treloar et al. 2022). Implementation of phylogenetic analysis in environments where there is potential for people living with HIV to be further targeted is likely to heighten existing sensitivities around data generation, sharing and use, particularly given the historical and socio-cultural burdens associated with HIV. If left unaddressed, this may perpetuate—or possibly even intensify— reluctance to get tested or seek support due to possible legal repercussions related to actual or potential transmission of HIV (Csete et al. 2023; Mehta et al. 2019). A number of Australian state-level public health reference laboratories already perform HIV whole genome sequencing for drug resistance inference. The delay in integrating HIV phylogenetic analysis into the larger public health response is therefore not reflective of its epidemiological limitations but rather of the need to adopt a cautionary approach that addresses legal, ethical, privacy, and logistical concerns. This has necessitated time for consultation and partnership-building with HIV communities and for coordinated regulatory responses across multiple levels of government (Commonwealth Government 2023). Recent restructuring within governments and public health organizations may also have contributed to delayed progress.

This article provides an overview of current regulatory frameworks designed to manage HIV transmission and examines the implications of those frameworks for the introduction of phylogenetic analysis as a surveillance tool, using Victoria, Australia, as a case study. Victoria has a population of 6.8 million, around 10,000 of whom are believed to be living with HIV, with 220 new diagnoses in 2023 (Australian Bureau of Statistics 2023; Victorian Government Department of Health 2024). Our focus on Victoria reflects ongoing research evaluating the potential utility of phylogenetic analysis as part of this jurisdiction’s public health response to assist with eliminating HIV infections (Taiaroa et al. 2024).

We begin by outlining Victoria’s historical and current approaches to regulating HIV transmission. We then consider phylogenetic analysis, its utility as a public health surveillance tool, and the possible implications of Victoria’s current regulatory approach. In the final section, we outline several regulatory considerations for policymakers if implementation proceeds. In doing so, we adopt a broad construction of regulation that acknowledges the role of other actors beyond state actors (e.g., health departments and law enforcement) that can (and should) have a role in regulation (Black 2002). This broad construction recognizes that regulation involves tools beyond law and government-issued policy, including guidelines and education (Black 2002), and highlights the interplay between different regulatory actors and tools. This approach also underscores the importance of having appropriate and tailored laws, policies, and guidance in place, coupled with meaningful community engagement and support, all of which have been identified as necessary for improved public health approaches to managing HIV transmission (Schuster et al. 2024). While we have narrowed our focus to Victoria, we note many jurisdictions in Australia and internationally adopt broadly similar approaches to infectious disease management. Hence, many of our observations are likely to have broader relevance.

## Regulatory Frameworks Relevant to HIV Transmission in Australia

Before outlining Victoria’s specific approach, we begin this section by outlining federal efforts to control and minimize HIV transmission, which apply to all Australian states and territories. At a federal level, overarching guidelines describe a staged public health approach to managing HIV transmission, intended to establish a consistent framework among all the states and territories (Commonwealth Government 2018). These guidelines, *inter alia,* set out a series of principles for managing transmission, assign roles and responsibilities, and outline a management framework (Commonwealth Government 2018). Moreover, a federal HIV taskforce—comprising representatives from the government, peak bodies, researchers, advocates, medical experts, and community organizations—has been charged with identifying HIV issues and trends to help end transmission (Commonwealth Government 2023).

Despite these federal efforts, much of the day-to-day management of HIV transmission rests with individual states and territories through the application of state-level legislation, policy, and public health initiatives. While approaches vary, jurisdictions broadly adopt a dual approach, involving both public health law (and policy) and criminal law. These two mechanisms serve distinct purposes. Public health measures—both legal and non-legal—are designed to safeguard the population (rather than just individuals) by managing and controlling the spread of infectious diseases (Carter 2016). Conversely, criminal law functions to respond to severe criminal conduct and serve as a deterrent (Carter 2016). Importantly, as discussed later in this article, these two approaches do not always operate in isolation and may intersect in practice.

For the remainder of this section, we focus specifically on Victoria’s approach to regulation. We begin with historical approaches, providing necessary context and tracing the evolution of the law, before focusing on current approaches. In setting out the current framework, we do not seek to be exhaustive and have necessarily confined our discussion. We acknowledge that other areas of law—such as migration, privacy, and newly enacted affirmative consent laws—may also be relevant to the implementation of phylogenetic analysis but fall outside the ambit of this article.

### Historical Approaches to Regulating HIV Transmission in Victoria

Early approaches to managing HIV transmission in Victoria embodied HIV exceptionalism— a phenomenon that arose partly because an HIV diagnosis at the time was considered akin to a death sentence, and tight regulation was seen as necessary. Even though medical science advanced, and preventative treatment options became increasingly available, the law failed to keep pace, resulting in a form of exceptionalism whereby legislative provisions specifically targeted individuals living with HIV. In Victorian criminal law, the now repealed section 19A of the *Crimes Act 1958* (Vic) made it an offence for a person to “intentionally cause[] another person to be infected with a very serious disease.” Unlike similar offences found in other Australian jurisdictions at the time, the legislative definition of “very serious disease” in Victoria applied solely to HIV (Carter 2016). Thus the Victorian offence although framed generally—couched in the terms “very serious disease”—in effect operated as an HIV-specific offence. 

Section 19A was introduced in 1993 following several armed robberies where victims were threatened with syringes containing blood (Legislative Assembly, 15 April 2015, 954). It carried a harsh maximum penalty of twenty-five years’ imprisonment—harsher than other offences targeting transmission of disease or causing serious injury, both of which attracted maximum penalties of twenty years (Legislative Assembly, April 15, 2015, 954). Opposition to section 19A was widespread (Carter 2020). This opposition stemmed from concerns about stigma, its potential to disincentivize the disclosure of HIV, and the fact that the penalties were disproportionate to the harm caused (Carter 2020). After years of criticism, the provision was finally repealed in 2015. The repeal reflected recognition that the law was out of step with society’s evolving understanding of HIV risk and was motivated by a desire to reduce stigma and discrimination, and promote equality (Legislative Assembly, April 15, 2015, 954).

HIV exceptionalism was also evident in public health law. For instance, special requirements attached to HIV testing prior to their removal in 2019, with their removal described as “a major step towards driving down stigma and discrimination” (Premier of Victoria 2019). Similarly, in 2022, amendments were made to further reduce stigma and discrimination by removing explicit mention of HIV^2^ in public health legislative provisions, including those relating to blood donations, tissue donations, and closure of courts and tribunals (*Public Health and Wellbeing Amendment Act 2022* (Vic).

### Current Approaches to Regulating HIV Transmission in Victoria

The current approach to regulating transmission in Victoria is thought to better reflect modern scientific and societal views. While HIV is no longer deemed a death sentence and most people living with HIV are not seen as a risk to public health, it has been suggested that the behaviour of a small number of individuals may contribute to transmission and pose a public health risk (Victorian Government Department of Health 2021). Accordingly, HIV transmission continues to be subject to regulation and oversight that relies on both a public health response and criminal law, both of which are discussed below.

#### Public Health Response

The prominent regulatory response to HIV transmission adopts a public health lens, engaging the legislative and administrative powers of the state via public health officials with the intention of safeguarding population health (Carter 2016). In Victoria, the primary regulatory tools include the *Public Health and Wellbeing Act 2008* (Vic) (“PHWA”) and the Victorian Guidelines for Managing HIV Transmission Risk Behaviours (2021), which are applied by the state’s Chief Health Officer (CHO).

The PHWA contains general provisions that apply to infectious diseases including HIV. Section 111 sets out guiding principles aimed at minimizing the spread of infectious diseases and identifying the shared responsibility of the state and community members. The legislation acknowledges personal responsibility—that a person who has or is suspected of having an infectious disease should take steps to eliminate or reduce transmission risk— while recognizing that others also hold responsibility for taking reasonable precautions to avoid contracting the disease.

Reflecting the administrative nature of the provisions, the PHWA grants the CHO a wide range of powers to manage and control infectious diseases, including testing and examination orders (s 113) and public health orders (s 117) that may require a person to take—or refrain from—specific actions to prevent, as far as possible, the infectious disease constituting a serious risk to public health. (Box 1 outlines the list of conditions that may be specified in public health orders.) The Act requires that, in cases where there are alternative measures available that are equally effective at minimizing a risk that a person poses to public health, the CHO chooses the measure that is least restrictive of the person’s rights (s 112). To issue an order, the CHO must believe the person has, or has been exposed to, an infectious disease and constitutes a serious risk to public health (ss 113,117). The CHO must believe that there was a reasonable attempt to provide the person with information about the effects of HIV on their health and its associated public health risk before an order is issued, unless it is impractical to do so (ss 113,117). The person subject to a public health order has a right to review the order through an internal review mechanism (via the CHO) or external review (via the Victorian Civil and Administrative Tribunal) (ss 121,122). The penalties associated with non-compliance are generally less severe and carry less social weight and stigma than those found in criminal prosecutions (Rush 2009; Scamell and Ward 2009). Nonetheless, public health measures can still be severe (e.g., detention), and, in rare cases, the criminal justice system can be engaged even when a person is being managed under a public health response.

**Box 1** List of Conditions Specified in Public Health Orders (s 117)

**Table:** 

- Participate in counselling, education, or other activities
- Undergo an assessment by a specified psychiatrist or neurologist
- Refrain from carrying out certain activities and behaviours (or only carry them out when specified conditions are met)
- Refrain from visiting a specified place (or class of place)
- Reside at a specified place of residence at all times or during specified times
- Notify change of name or place of residence
- Submit to the supervision of a nominated person (e.g., by attending meetings, receiving visits, or providing information)
- Receive specified prophylaxis
- Undergo specified pharmacological treatment
- Submit to being detained or isolated (or both).

HIV-specific guidelines issued by the Victorian Government work in concert with the PHWA. These guidelines provide content to the legislative provisions and operationalize the law by setting out a framework for managing the behaviours of people living with HIV. Like the PHWA, the guidelines seek to strike the right balance between mitigating the risk of HIV to the community and protecting the rights of individuals. Accordingly, the guidelines are only applied when there is a perceived risk to the public and do not apply in cases where a person has taken reasonable steps to minimize transmission. While the guidelines do not offer an exhaustive list of what would constitute reasonable steps, they do provide some examples. These include having a sustained undetectable viral load (less than 200 copies/mL), consistent condom use with lubricant, consistent disclosure of HIV status to sexual partners, and seeking confirmation from sexual partners that they are taking PrEP before engaging in sex (Victorian Government Department of Health 2021).

**Figure 1:**
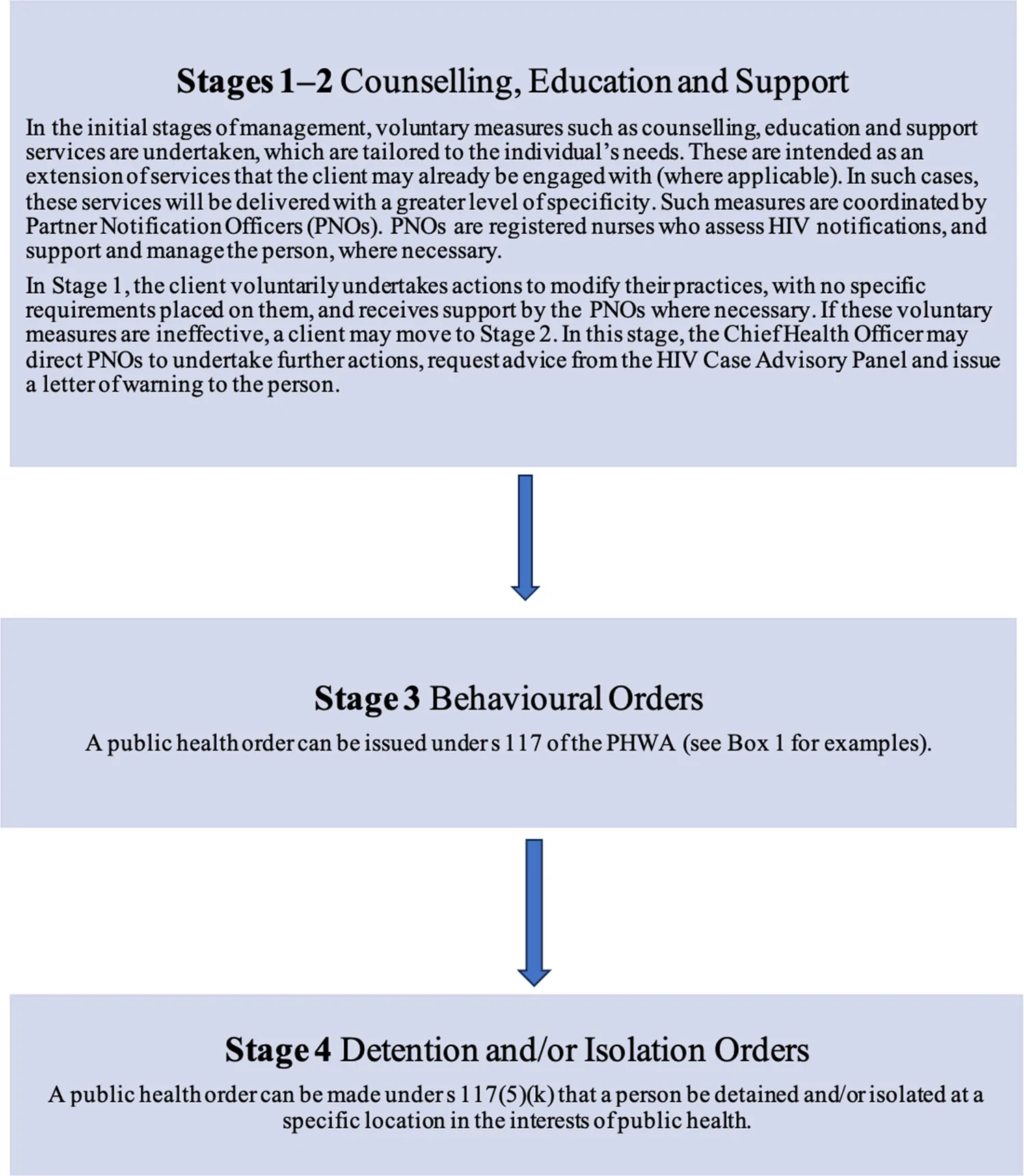
Four-Stage Approach to HIV Management (The figure was created by the authors, drawing on *Victoria’s Guidelines for Managing HIV Transmission Risk Behaviours* (Victorian Government Department of Health 2021).)

The framework employs a four-stage approach (see figure 1 for an overview), establishing a hierarchy of measures (non-legislative and legislative) to manage HIV transmission. In most cases, the framework operates primarily through escalation, employing less coercive measures aimed at modifying behaviours (e.g., education, counselling, and support) in the first instance, followed by more coercive practices (public health orders, including behavioural, detention, and/or isolation orders). These more coercive measures are intended to be reserved for exceptional cases where less coercive measures have been exhausted or deemed ineffective. However, it is also recognized that measures may be applied in a non-sequential order in exceptional cases (or when required) (Victorian Government Department of Health 2021, 20).

While the public health and criminal law approaches serve distinct functions, they do not operate in isolation. In exceptional cases, where a person fails to comply with public health orders, or where risky HIV transmission practices are continued with clear intent to cause harm—or with serious disregard for the wellbeing of others—a referral to police may occur (Victorian Government Department of Health 2021, 26).

#### Criminal Law

While Victoria’s approach to regulating the transmission of HIV is predominately public health based, criminal law continues to play a regulatory role beyond cases of non-compliance with public health orders. Although HIV-specific offences no longer exist, general criminal provisions may still apply. The use of general criminal law rather than HIV-specific offences receives qualified endorsement by various United Nations bodies, alongside strong caution that criminal law should only be employed in exceptional circumstances where there is actual and intentional transmission of HIV (UNAIDS Reference Group on HIV and Human Rights, 2023).

While there are potentially a range of criminal offences that may apply to HIV transmission, in practice, the offences relied on fall into two broad categories. These are assault offences, which depend on HIV transmission having occurred, and endangerment offences, which apply to potential rather than actual transmission. Table 1 provides an overview of relevant Victorian criminal provisions that may be (and, in some cases, have been) applied in the context of HIV.

**Table 1:** General Criminal Offences

Section number	Type of offence	Offence description	Offence	Maximum penalty
16	Assault	Causing serious injury^a^ intentionally	A person who, without lawful excuse, intentionally causes serious injury to another person is guilty of an indictable offence.	20 years
17	Assault	Causing serious injury recklessly	A person who, without lawful excuse, recklessly causes serious injury to another person is guilty of an indictable offence.	15 years
24	Assault	Negligently causing serious injury	A person who by negligently doing or omitting to do an act causes serious injury to another person is guilty of an indictable offence.	10 years
22	Endangerment	Conduct endangering life	A person who, without lawful excuse, recklessly engages in conduct that places or may place another person in danger of death is guilty of an indictable offence.	10 years
23	Endangerment	Conduct endangering persons	A person who, without lawful excuse, recklessly engages in conduct that places or may place another person in danger of serious injury is guilty of an indictable offence.	5 years

^a^Injury is inclusive of physical injury which includes “infection with a disease.” See section 15 of the *Crimes Act 1958* (Vic).

Like the more coercive measures of the public health response, engagement with the criminal justice system is intended to occur only in exceptional cases and as a measure of last resort, consistent with the federal and state guidelines previously discussed (Commonwealth Government 2018; Victorian Government Department of Health 2021). In this sense, criminal law can be characterized as a “mop up strategy” used when public health interventions have been ineffective (Rush 2009). However, as will be further discussed below, concerns have been raised that criminal law is being applied outside of these circumstances and engaged independently of the public health response.

To secure a conviction, the prosecution must establish criminal responsibility. To prove intention or recklessness, the requisite mental element must be established—the person must have known that they have HIV (Woodroffe 2009). This contrasts with the public health response, which recognizes that preventing transmission is a shared responsibility and that every individual, irrespective of their HIV status, has a responsibility to engage in safer sex practices to reduce transmission, including by maintaining a low viral load (in the case of a person living with HIV) or taking preventive measures (in the case of a person not living with HIV) (Woodroffe 2009). Instead, criminal laws inadvertently punish those who are aware of their HIV-positive status, even though this cohort is much less likely to transmit HIV (Hall et al. 2012). Such an approach may encourage HIV status ignorance (strategic or otherwise) and may serve as a deterrent to testing.

## Nature of HIV Phylogenetic Analysis and Implications for Implementation

Having considered existing regulatory approaches, we now consider how this may impact any potential future implementation of phylogenetic analysis. We begin this section by providing an overview of phylogenetic analysis and its potential use as a public health surveillance tool, before identifying limitations that may impact implementation. Although our focus here is on HIV, such an analytical tool has been (and could further be) used in the context of other infectious diseases; hence, some of our observations may be generalizable.

At present, HIV surveillance in Australia relies on traditional epidemiological data to monitor transmission. Since the early 2000s, genetic sequence analysis has been used to identify the subtype of HIV with which an individual is infected and to identify any drug-resistant mutations that may inform the optimal use of antiviral therapy. These genetic sequence data can be repurposed for phylogenetic analysis. Analysing these sequences in combination with clinical and behavioural data can reveal transmission patterns, detect emerging resistance mutations, and identify possible linkages among cases, thereby contributing to more effective public health responses. This integration of traditional data with epidemiological analysis allows for a more comprehensive understanding of HIV spread and enhances the effectiveness of current treatment strategies within specific populations (Taiaroa et al. 2024). This is possible because of HIV’s ability to evolve rapidly, creating significant viral diversity within infected individuals and populations across time, allowing for the identification of real-time HIV transmission clusters and people who are at a high proximal risk of HIV transmission (Schairer et al. 2019). Unlike some European and North American jurisdictions, Australia does not currently use phylogenetic analysis for HIV surveillance (HIV Justice Worldwide 2021). However, this analytical tool is being used in other contexts in Australia, including for the prevention and control of other conditions such as tuberculosis, foodborne illnesses, and SARS-CoV-2 (Meumann et al. 2021; Parker et al. 2023; Roulis et al. 2015; Seemann et al. 2020).

Phylogenetic analysis can provide a robust understanding of disease distribution and spread —such as by identifying transmission hotspots—yielding benefits for individual and community health, and can serve to complement existing approaches (Chung et al. 2019; HIV Justice Network 2022; Obeng et al. 2024). It can also assist with priority setting and help to inform the design and implementation of policy and interventions, particularly in relation to harder-to-reach and harder-to-detect groups (Coltart et al. 2018; Jamrozik et al. 2023; German et al. 2017). International evidence supports its utility in the identification of priority clusters, partner notification, PrEP allocation, social engagement, and the development of public health campaigns, such as those linked to care and treatment (Beltrami et al. 2018; Obeng et al. 2024; Oster et al. 2021). Despite such benefits, the nature of the data and the current regulatory framework present barriers to implementation if concerns are not appropriately addressed.

### Features of Phylogenetic Data

One concern about the use of phylogenetic analysis relates to the nature of its data. Phylogenetic analysis cannot conclusively determine directionality of transmission (Barré-Sinoussi et al. 2018; Bernard et al. 2007; Boyd et al. 2016; Brooks and Sandstrom 2013; Chung et al. 2019; Cranston 2020; Kidd 2016; Sandset 2020; Taylor and Sapién 2021). This is because phylogenetic analysis methods, at present, generally cannot rule out third-party infections or complex transmission patterns involving epidemiologically linked variants (Abecasis et al. 2018; Barré-Sinoussi et al. 2018; Boyd et al. 2016; Kidd 2016). However, while directionality cannot be determined, information about the level of genetic relatedness between samples is possible (Brooks and Sandstrom 2013; Sandset 2020). Consequently, in cases where phylogenetic analysis fails to demonstrate a close association between strains, it can be used to definitively exclude individuals as being the source of infection (Abecasis et al. 2018; Barré-Sinoussi et al. 2018; Bernard et al. 2007; Brooks and Sandstrom, 2013).

The accuracy of results, however, depends on the completeness of the dataset and the representativeness of samples, as well as the analytical methods and algorithms used to reconstruct the phylogeny (Boyd et al. 2016; Obeng et al. 2024). Accordingly, while useful for population-based interventions, phylogenetic data must be applied with caution when considering person-to-person transmission. To enhance reliability, such data must be interpreted alongside additional demographic and epidemiological data (Barré-Sinoussi et al. 2018; Bernard et al. 2007; Dawson et al. 2020).

Understanding the technology and interpretative limits of phylogenetic analysis is particularly important where data is used to justify public health orders or criminal prosecution. In criminal trials, it has been argued that the lack of safeguarding requirements to ensure that experts draw on reliable or scientifically valid evidence is of concern, given that expert opinions are admissible in Australian courts (Kidd 2016). Further, even if the data is presented accurately, whether the legal threshold has been satisfied ultimately rests with judges and jurors. Therefore, if the data is not translated and fully explained in a way that ensures such personnel can fully appreciate its nature, there is risk of misinterpretation—for example, inferring directionality—leading to flawed application of the relevant legal standard (Perin 2021).

### Concerns Pertaining to Weaponizing Data

Concerns about implementation also arise due to insufficient community consultation, potential increased stigma, privacy issues, and the possibility of phylogenetic data being weaponized—for example, to prosecute or to hand down coercive public health orders—under the guise of public health, particularly given existing links between health authorities and law enforcement (HIV Justice Worldwide 2021). Indeed, much of the apprehension relates to fears of greater state control, objectification, enforcement of morality, and punishment of social vulnerability (Bernard et al. 2020; Buchbinder et al. 2020; HIV Justice Network 2022; Shook et al. 2022). Under current regulatory frameworks, individuals living with HIV may be subject to general criminal offences that imply culpability, despite evidence showing that criminalization does not significantly reduce transmission (Jürgens et al. 2009). Incarceration may even exacerbate risk due to the prevalence of sexual activity, intravenous drug use, and limited access to preventive measures (such as condoms) in detention settings (Jürgens et al. 2011).

The current framing of criminal laws means culpability is assigned to the person living with HIV due to their ability to transmit the virus, leaving them particularly vulnerable—despite evidence that people who know their status are less likely to transmit the virus (Hall, Holtgrave and Maulsby 2012). This means that, in contrast to public health law and associated guidelines, criminal law does not recognize the responsibilities of sexual partners to take preventive measures to reduce the risk of HIV transmission. In some Australian cases, accusations have reportedly been used to threaten, blackmail, or exert coercive control over HIV-positive individuals (NAPWHA 2023).

In a similar vein, regulatory frameworks that enable the targeting and punishment of those living with HIV have reportedly resulted in distrust among members of the HIV community and, in some cases, have deterred people from testing or treatment (Kilty and Bogosavljevic 2018; Schairer et al. 2017; The Lancet 2018). Findings from an Australian study drawing on cross-sectional data found that “the criminalisation of HIV transmission, likely reinforced by ongoing media reporting around this issue, has contributed to considerable anxiety among people living with HIV in Australia” (Bourne et al. 2022, 4). While both public health and criminal law can target individuals and employ coercive measures, greater concern has been expressed in relation to criminal law.

Such fears are not unfounded. Indeed, there is evidence of prosecutions and other actions against people living with HIV being advanced by means of spurious scientific claims (Cranston 2020). In Australia, prosecution under assault and endangerment offences has occurred across jurisdictions, resulting in an estimated fifty-three prosecutions to date (HIV Justice Network 2023a).^3^ It is also notable that HIV is the only blood-borne virus or STI that is, in practice, prosecuted under these general criminal laws (Commonwealth Government 2023).

A further concern is the tendency for specific sub-populations to be disproportionally targeted by prosecution and public health investigations, including sex workers, gay and bisexual men, drug users, transgender communities, Indigenous people, immigrants, and racial minorities—a trend observed both within Australia and internationally (Csete et al. 2023; Carter 2020; Jamrozik et al. 2023; HIV Justice Network 2022; Taylor and Sapién 2021). Targeted prosecutions (and the associated stigma and discrimination) are likely to prevail, even with the increasing availability and efficacy of ART in reducing transmission, given disparities in access to ART and discontinuation rates within these groups (Carter 2020; Medland et al. 2023). The ability of phylogenetic analysis to identify clusters is likely to serve to further stigmatize these groups and heighten anxiety (Molldrem and Smith 2024).

## Recommendations and Considerations for Implementation

This final section builds on the preceding sections and offers regulatory recommendations and considerations (see Box 2). While the focus is on phylogenetic analysis, some of the proposals may also be applicable outside this context.

**Box 2** Regulatory considerations and recommendations if phylogenetic analysis is introduced for the purposes of HIV public health surveillance

**Table:** 

**Law reform** 1. Review the operation of laws that currently criminalize or otherwise target HIV transmission, and consider whether reform is needed (this may include a formal inquiry).
**Procedural and practice considerations** 1. Have policies in place to ensure only those who are appropriately qualified and understand the nature of phylogenetic analysis (including its limitations) can analyse and report on data arising from HIV phylogenetic analysis. 2. Have communication and reporting standards in place for reporting on (or otherwise relying on) data arising from HIV phylogenetic analysis to ensure that any interpretation (and subsequent actions taken) accounts for context. 3. Have policy directives or a memorandum of understanding in place to ensure that police/prosecutors consult with (or obtain approval from) Health Department personnel before prosecuting. 4. Appoint an external oversight body to scrutinize the use of phylogenetic analysis for HIV surveillance during the early stages of implementation and potentially on an ongoing basis. This could take the form of prospective oversight, retrospective oversight, or a combination approach.
**Guidance and education** 1. Develop and make available education and guidance (factsheets, guidelines, etc) on phylogenetic analysis and HIV. 2. In addition to general education and guidance, ensure tailored education and guidance are developed for police, prosecutors, judges, the HIV community, Health Department staff (including data analysts), policymakers, health professionals, expert witnesses, and the media.
**Community engagement and research** 1. Ensure meaningful engagement and consultation are carried out with the HIV community before implementation and sustained post-implementation. 2. Continue efforts to address HIV-related stigma at the individual and structural level, alongside other regulatory interventions. 3. Continue (and, where possible, increase) efforts to address HIV transmission through: (a) increasing accessibility of testing, diagnosis, care, and treatment, targeting appropriate sub-populations where necessary; and (b) delivering targeted community awareness-raising and health promotion campaigns. 4. Continue to build a solid evidence base around HIV transmission, including evaluating the effectiveness of the strategies and surveillance systems intended to reduce HIV transmission.

Acknowledging the concerns relating to criminalization covered in the previous section—and the strong preference, especially in the HIV-positive community, for the decriminalization of HIV transmission—some argue that dismantling stigmatizing and threatening laws is a necessary precondition for ethical implementation of phylogenetic analysis (Cranston 2020). However, leaving open the possibility of engaging the criminal law in exceptional cases and as a last resort could be justifiable and is consistent with international recommendations (UNAIDS Reference Group on HIV and Human Rights 2023). There is also some evidence to suggest that criminal law can serve to persuade people living with HIV who have fallen out of care to re-engage (Buchbinder et al. 2020). Nevertheless, there is evidence to suggest that the criminal law is being used outside these narrow circumstances.

The potential for current regulation to deter testing or engagement with care may undermine the public health benefits that flow from introducing phylogenetic analysis for the purposes of public health surveillance. This provides a compelling case for law reform or, at minimum, a formal inquiry (e.g., law reform commission inquiry or parliamentary inquiry) into the current application of criminal law in HIV cases. Such a review would allow dedicated examination rather than reliance on external reports that may be limited in scope or approach. If a formal inquiry proceeds, it should also examine the use of public health orders in HIV cases and include meaningful community consultation.

Full decriminalization may not be possible or desirable in all cases, but reform to exclude negligent or reckless transmission from criminal prosecution —reserving criminal processes only for cases where intentional transmission is proven to the appropriate standard—may be advantageous. This would retain criminal law as a deterrent, while limiting its use to egregious cases.

Given the difficulties that arise with achieving such reform in Australia, and recognizing the absence of HIV-specific offences, we offer recommendations for additional regulatory measures, drawing on the broad construction of regulation we set out in the beginning of the article, which acknowledges that regulation extends beyond law and is carried out by both state and non-state actors (Black 2002).

The three broad forms of regulation that we believe should be considered are: (1) procedural and practice considerations, (2) education and guidance, and (3) community engagement and research. These forms of regulation complement existing regulatory frameworks and may be applied in combination. Some may still be relevant even if phylogenetic analysis is not implemented.

### Procedural and Practice Considerations

Minimizing risks associated with phylogenetic analysis requires establishing systems that guard against misuse. This includes ensuring those analysing and reporting on the phylogenetic analysis data are appropriately qualified and intimately familiar with the application of phylogenetic analysis and its limitations (Barré-Sinoussi et al. 2018). It is equally important that the capabilities of the technology and the limitations of the data are cogently and accurately communicated when reporting on or otherwise relying on the data, so any interpretation (and subsequent actions) are appreciated in context. The establishment of minimum standards for reporting data (including by Health Departments and media) is essential.

Consideration should also be given to establishing systems to ensure that police are required to consult (or receive approval from) appropriately qualified Health Department personnel before they take action against any HIV-related complaints. This could be achieved through policy directives (as suggested by federal guidelines) or other forms of agreements such as memorandums of understanding.

If this form of surveillance is introduced, it should be subject to external oversight, particularly during initial implementation, to ensure consistency with public health objectives and limitations on coercive enforcement. Such oversight could be carried out by a dedicated panel, independent of the Department of Health and law enforcement, comprising multiple personnel from various disciplines and community representation. The existing HIV Case Advisory Panel reflects such a composition and currently provides the CHO with advice, where appropriate, pertaining to public health orders and referrals to police, in the rare cases where this occurs. There may be scope for the panel’s terms of reference to be revised to include a role in reviewing the use of phylogenetic data.

If such a panel is adopted, consideration should be given to how such a panel will provide oversight. Prospective oversight is one option, whereby approval is sought to use data before any prosecution takes place (similar to current consultation processes that occur when a person with HIV is managed under the guidelines). Retrospective oversight is an alternative whereby each prosecution using phylogenetic data is reviewed by a panel or auditor (similar to how quality improvement audits are carried out) to ensure it is justified and consistent with principles set out in the guidelines and to ensure that the data is not being overstated or misinterpreted. Alternatively, a combination of prospective and retrospective oversight could be used, as is currently the case in Victoria in the voluntary assisted dying context (White et al. 2021).

### Guidance and Education

Guidance and education in relation to phylogenetic analysis and HIV more broadly will be essential if this tool is introduced. This should cover, *inter alia*, the limitations of phylogenetic analysis in identifying transmission directions, and the current mortality rate of HIV and treatment options to provide a realistic picture of costs and benefits. Information should be widely communicated to the community and directed towards influencing prosecutors and decision-makers, to prevent inappropriate use of coercive measures counterproductive to public health.

Targeted guidance and education is likely to be most effective and should include law enforcement actors—police, prosecutors, and judges—given the immense power they hold regarding prosecution (Galletly et al. 2019; German et al. 2017). However, it is also crucial that other relevant actors are also informed and educated as necessary, including, but not limited to, the HIV community, Health Department staff (including any data analysts), policymakers, health professionals and other personnel acting as expert witnesses. Given potential implications for clinical practice, it is important that health professionals are adequately educated on phylogenetic analysis. For example, health professionals may be asked questions during a consultation about how phylogenetic data is used in the clinical and public health context, and hence may need to know where to direct patient enquiries to, or may be required to interpret results in order to recommend more appropriate treatment options (e.g., in the case of a drug-resistant strain of HIV). Therefore, ensuring that health professionals have appropriate guidance and education to navigate such clinical encounters is imperative.

Internationally, there have been efforts to develop guidance and education in various forms, including prosecution guidelines, consensus statements, policies, policy recommendations, education modules, and briefing papers. These all serve the purpose of steering and guiding behaviour and have been produced by bodies including the United Nations, scientific organizations, and community and advocacy groups, such as HIV Justice (Barré-Sinoussi et al. 2018; HIV Justice Network n.d.).

Within the Australian context, there is also evidence of such efforts. For instance, in 2016, leading HIV clinicians and scientists published a consensus statement to summarize current scientific evidence in order to facilitate just outcomes in Australian criminal cases (Boyd et al. 2016). Similarly, the HIV/AIDS Legal Centre has produced a guide for legal practitioners in New South Wales (HALC 2009), and HIV Online Learning Australia has made a community-oriented educational paper on HIV phylogenetics (Di Giallonardo et al. 2023).

Another important target for education and guidance is the media. As noted above, stigma associated with criminalization is often exacerbated by media reporting (Bourne et al. 2022). In the context of phylogenetic analysis, this is especially problematic, as suggested by a recent study by Molldrem and Smith (2024) among stakeholders in the United States, which found that public communication and media reporting around emerging clusters could contribute to stigmatization and harassment.

For completeness, it is worth noting that there are mixed views on the effectiveness of these “softer”—educative—forms of regulation. For instance, Canada and the United States remain hotspots for criminalization (McClelland et al. 2020) despite this approach being out of step with recommendations of an international consensus statement (Barré-Sinoussi et al. 2018). HIV Justice Network, however, note that the statement has had benefits. Indeed, it has been instrumental in successful defences in court, useful in advocacy campaigns, and helped support education and awareness-raising initiatives (HIV Justice Network 2023b). Speaking more broadly about these educative approaches to regulation, Weait has argued that it is impossible to make conclusions about their impact with any confidence (Weait 2019). However, he does concede, where success has been achieved, that it is unlikely this would have occurred (or occurred as quickly) without this aggregate level of activity (Weait 2019).

### Community Engagement and Research

Meaningful engagement with the HIV community prior to implementing surveillance measures is essential to reduce fear, address distrust, and avoid people from being deterred from testing. This would help identify any concerns the community may have and help ensure future initiatives address (or at least abate) such concerns. Outside this, efforts to try and address the perennial issues of HIV-related stigma, at the individual and structural level, should continue in parallel. However, such efforts are unlikely to be solutions in and of themselves, as evidenced by the prevailing stigma present within the community reflecting their ineffectiveness to date. Efforts to increase access to testing, diagnosis, care, and treatment, accompanied by targeted community awareness-raising and health promotion campaigns (particularly among vulnerable sub-populations) also remain critical. Although it is acknowledged, with respect to the latter point, implementing phylogenetic analysis would assist in targeting interventions.

Finally, continued development of a solid evidence base around HIV transmission in Australia is imperative. Future research should continue to explore the efficacy of different strategies used to minimize HIV transmission and examine the HIV community’s views on their application, including surveillance systems should they be introduced.

## Conclusion

Utilizing phylogenetic analysis as part of the public health response to HIV transmission may yield benefits but also raises several legal and ethical concerns, particularly under the current regulatory framework where criminal prosecution remains possible. If it is determined that phylogenetic analysis would support Victoria’s approach to HIV transmission management, careful consideration must be given, before implementation, to how this is best regulated. This should include assessment of necessary reforms to existing regulatory frameworks and identification of what additional forms of regulation may be required. Such consideration should extend beyond law and policy (across state and federal levels) to include procedural and practice elements, education and guidance, community engagement, and research.

## Data Availability

Not applicable to this research.

## Footnotes

1 This concept has often been referred to as Undetectable=Untransmittable (U=U).

2 Note that such amendments also removed mention of Hepatitis C. Further, while these infectious diseases are no longer explicitly mentioned in primary legislation, they do exist in subordinate legislation in the form of regulations.

3 There are no publicly available data on the prosecution of criminal offences created by public law (Carter 2020).
